# Proteins Associated with Phagocytosis Alteration in Retinal Pigment Epithelial Cells Derived from Age-Related Macular Degeneration Patients

**DOI:** 10.3390/antiox11040713

**Published:** 2022-04-05

**Authors:** Audrey Voisin, Afsaneh Gaillard, Anaïs Balbous, Nicolas Leveziel

**Affiliations:** 1 Laboratoire de Neurosciences Expérimentales et Cliniques, Equipe Thérapie Cellulaire dans les Pathologies Cérébrales, INSERM, Université de Poitiers, F-86073 Poitiers, France; afsaneh.gaillard@univ-poitiers.fr (A.G.); anais.balbous.gautier@univ-poitiers.fr (A.B.); nicolas.leveziel@chu-poitiers.fr (N.L.); 2 CHU Poitiers, F-86021 Poitiers, France

**Keywords:** age-related macular degeneration, atrophic AMD, exudative AMD, iPSC-RPE phagocytosis, RNA-seq

## Abstract

Age-related macular degeneration (AMD) is partially characterized by retinal pigment epithelial (RPE) cell dysfunction. This study focused on phagocytosis activity and its involvement in AMD. Phagocytic activity was analyzed by flow cytometry using porcine photoreceptor outer segment (POS) and fluorescent beads in basal and under oxidative stress condition induced by Fe-NTA in fifteen hiPSC-RPE cell lines (six controls, six atrophic AMD and three exudative AMD). Oxidative stress exposure inhibited phagocytosis in the same manner for control, atrophic AMD (AMDa) and exudative AMD (AMDe) cell lines. However, altered phagocytosis in basal condition in hiPSC-RPE AMDa/e was observed compared to control cell lines. Gene expression after 3 or 24 h of POS incubation was analyzed by RNA-Seq based transcriptomic profiling. Differential gene expression was observed by RNA seq after 3 and 24 h POS exposure. We have focused on the genes involved in mTOR/PI3K-AKT/MEK-ERK pathway. We investigated differences in gene expression by analyzing the expression levels and activity of the corresponding proteins by Western blot. We showed the involvement of three proteins essential for phagocytosis activity: fak, tuberin and rictor. These findings demonstrate that hiPSC-RPE AMDa/e cells have a typical disease phenotype characterized by alteration of the main function of RPE cells, phagocytosis activity.

## 1. Introduction

Age-related macular degeneration (AMD) is the first cause of blindness in the elderly population in developed countries, representing almost 15% of causes of blindness among adults aged ≥ 50 years in Western Europe [1]. With progressive aging of the population, the number of people with AMD in 2040 is projected to be 288 million [2]. The exudative form of AMD (AMDe) is characterized by choroidal neovascularization with subsequent bleeding and fluid exudation into the retina, while the atrophic form (AMDa) is characterized by retinal pigment epithelium (RPE) apoptosis associated with choriocapillaris atrophy and overlying photoreceptors degeneration [3,4]. AMD is a multifactorial complex disease, the major risk factors for both forms being ocular factors (age-related maculopathy) and genetic and environmental factors such as smoking [5,6]. Somatic cells derived from induced pluripotent stem cells (iPSC) can represent a useful source of an in vitro cellular model for diseases when derived from affected individuals, particularly in the cases of scarce animal or appropriate cellular models [7,8,9]. In this context, RPE cells derived from healthy and AMDa/e patients were previously obtained [10]. RPE cells are highly polarized and pigmented monolayer cells essential for normal vision [11]. RPE cells derived from individuals suffering from AMDa express a typical disease phenotype in a pro-oxidant environment when compared to hiPSC-RPE obtained from healthy individuals. hiPSC-RPE AMDa cells are more sensitive to iron exposure and have presented with dysregulation of lysosomal enzyme activities under oxidative stress condition [10]. Many studies have reported that RPE cell alterations induced by aging (i.e., mitochondrial damages, lysosomal dysregulation, accumulation of lipofuscin….) may play a role in the pathophysiology of this disease [11,12,13]. Oxidative stress is possibly one of the most critical factors able to induce pathological cascade leading to AMD development [14]. In our previous study, we demonstrated that hiPSC-RPE cells derived from AMD patients are more sensitive to iron exposure and dysregulate lysosomal enzyme activities under oxidative stress conditions [10]. Lysosomal activity is required for the digestion of photoreceptor outer segments (POS), the last stage of RPE phagocytosis [15,16]. RPE cells are specialized phagocytes playing a critical role in POS renewal [17,18]. Any imbalance in POS phagocytosis by RPE cells causes debris accumulation over time, ultimately harming photoreceptors [14,19]. RPE phagocytosis dysfunction is observed in the Royal College of Surgeons rats, an animal model of recessively inherited retinal degeneration [20], leading to blindness within a few weeks postnatal [21]. Since the last decade, some evidence has suggested that oxidative stress and free radical damage in the RPE lead to impaired RPE phagocytic function and underlie AMD pathogenesis [22]. In this context and in line with previous studies, we aimed to better understand the phagocytosis impairment observed in our AMD model derived from iPSC.

Age-related macular degeneration (AMD) is the first cause of blindness in the elderly population in developed countries, representing almost 15% of causes of blindness among adults aged ≥ 50 years in Western Europe [1]. With progressive aging of the population, the number of people with AMD in 2040 is projected to be 288 million [2]. The exudative form of AMD (AMDe) is characterized by choroidal neovascularization with subsequent bleeding and fluid exudation into the retina, while the atrophic form (AMDa) is characterized by retinal pigment epithelium (RPE) apoptosis associated with choriocapillaris atrophy and overlying photoreceptors degeneration [3,4]. AMD is a multifactorial complex disease, the major risk factors for both forms being ocular factors (age-related maculopathy) and genetic and environmental factors such as smoking [5,6]. Somatic cells derived from induced pluripotent stem cells (iPSC) can represent a useful source of an in vitro cellular model for diseases when derived from affected individuals, particularly in the cases of scarce animal or appropriate cellular models [7,8,9]. In this context, RPE cells derived from healthy and AMDa/e patients were previously obtained [10]. RPE cells are highly polarized and pigmented monolayer cells essential for normal vision [11]. RPE cells derived from individuals suffering from AMDa express a typical disease phenotype in a pro-oxidant environment when compared to hiPSC-RPE obtained from healthy individuals. hiPSC-RPE AMDa cells are more sensitive to iron exposure and have presented with dysregulation of lysosomal enzyme activities under oxidative stress condition [10]. Many studies have reported that RPE cell alterations induced by aging (i.e., mitochondrial damages, lysosomal dysregulation, accumulation of lipofuscin….) may play a role in the pathophysiology of this disease [11,12,13]. Oxidative stress is possibly one of the most critical factors able to induce pathological cascade leading to AMD development [14]. In our previous study, we demonstrated that hiPSC-RPE cells derived from AMD patients are more sensitive to iron exposure and dysregulate lysosomal enzyme activities under oxidative stress conditions [10]. Lysosomal activity is required for the digestion of photoreceptor outer segments (POS), the last stage of RPE phagocytosis [15,16]. RPE cells are specialized phagocytes playing a critical role in POS renewal [17,18]. Any imbalance in POS phagocytosis by RPE cells causes debris accumulation over time, ultimately harming photoreceptors [14,19]. RPE phagocytosis dysfunction is observed in the Royal College of Surgeons rats, an animal model of recessively inherited retinal degeneration [20], leading to blindness within a few weeks postnatal [21]. Since the last decade, some evidence has suggested that oxidative stress and free radical damage in the RPE lead to impaired RPE phagocytic function and underlie AMD pathogenesis [22]. In this context and in line with previous studies, we aimed to better understand the phagocytosis impairment observed in our AMD model derived from iPSC.

## 2. Materials and Methods

### 2.1. Patients and hiPSC-RPE Cell Lines

Individuals included in the control group (6 cell lines, 62.8 ± 16 y/o) were patients with normal fundus examination and no history of visual impairment. Patients included in the AMDa group (6 cell lines, 77.5 ± 7 y/o) presented a wide range of atrophy observed on fundus examination and on optical coherence tomography. Patients included in the AMDe group (3 cell lines, 89 ± 8 y/o) had a particularly severe form of exudative AMD, also called type 3 choroidal neovascularization. The hiPSC-RPE Control and AMDa cell lines used in this study were previously characterized [9,10]. Briefly, iPSC derived from an AMDe patient were reprogrammed from peripherical blood mononuclear cells by nucleofection of Yamanaka factors and were differentiated into RPE cells by spontaneous protocol. The hiPSC-RPE cells were cultured with DMEM High Glucose supplemented with 4% KSR (Invitrogen, Waltham, MA, USA; Thermo Fischer Scientific, Waltham, MA, USA). Medium was changed twice a week. The hiPSC-RPE cells were passaged every 3 weeks up to two passages.

### 2.2. Iron Treatment and Induction of Oxidative Stress

hiPSC-RPE cells (3 weeks after P2) were treated for 24 h with FeCl3-sodium nitrilotriacetate (Fe-NTA) at final concentrations of 5 to 20 mM as previously described [9].

### 2.3. Isolation of Porcine POS

POS were isolated according to established protocols from porcine eyes [23]. Briefly, extracted retinas were homogenized in phosphate buffer containing 20% sucrose (SigmaAldrich, Saint Louis, MO, USA), 20 mM tris acetate pH 7.2 (Sigma), 2 mM MgCl_2_ (Sigma), 10 mM glucose (Sigma) and 5 mM taurine (Sigma) in the dark. The suspension was shaken vigorously for 2 min and carefully overlayed on the top of a discontinuous sucrose density gradient (25–60%) and centrifuged at 106,000× *g* for 48 min at 4 °C. POS fraction, identified as a single orange band, was collected and washed 3 times with tris acetate buffer and pelleted by centrifugation at 3000× *g* for 10 min at 4 °C. POSs were labeled with fluorescein-5-isothiocyanate (FITC, Invitrogen) by incubation in DMEM containing 0.5 mg/mL FITC for 1 h 30 min at room temperature in the dark and on agitation. After labeling, POSs were washed, counted and resuspended in growth medium supplemented with 2.5% sucrose; they were then counted and stored at −80 °C.

### 2.4. Analysis of Phagocytosis by Flow Cytometry

The hiPSC-RPE cells were incubated for 3 or 24 h with fluorescent carbonylate-modified microspheres (1 or 2 µm, ThermoFischer) or FITC-POS. The cells were then washed 3 times with PBS and maintained in 1 mL of PBS-2% SVF culture medium. Fluorescence (505/515 nm) was analyzed with FACS Verse (BD Biosciences, Franklin Lakes, NJ, USA) and FlowJo^®^ software (BD Biosciences)).

### 2.5. Total RNA Sequencing

RNA from 0 (unchallenged), 3 and 24 h POS exposure hiPSC-RPE (6 control, 6 AMDa, 3 AMDe) were isolated with RNeasy micro kit (Qiagen, Hilden, Germany), following manufacturer’s protocol. Purity and concentration of RNA were determined by a ND-1000 spectrophotometer NanoDrop (Thermo Fischer Scientific). RNA sequencing was performed using the TruSeq Stranded mRNA Library Prep Kit (Genoscreen, Lille, France). Each cDNA library was generated and run separately on two lanes of the HiSeq 4000 (Illumina, San Diego, CA, USA) in order to obtain a minimum of 40 million paired-end reads per sample. To prepare the reads for alignment, the sequencing adapters and other low-quality bases were clipped. Raw reads were processed and aligned with the human genome assembly reference GRCh38.p13 using bowtie2 v2.3.4.3 software. Data normalization and differential expression (DE) analyses were performed with RSEM v1.3.1 and EBSeq software [24], with a false discovery rate (FDR) < 0.05 considered as statistically significant. Fold change values were replaced by log_2_ values in order to make the variation more noticeable. A fold-change criterion was employed with the mean fold change between two conditions to be ≥2.5 or ≤−2.5 [25]. Metascape software was used for Gene Ontology (GO) annotation. Volcano plot performed with Prims software was used to visualize the DE gene between hiPSC-RPE Control, AMDa and AMDe cells.

### 2.6. Western Blot

The hiPSC-RPE cells were incubated for 3 or 24 h with POS and lysed in the extraction buffer RIPA (Sigma) with protease and phosphatase inhibitor cocktail (Sigma). Briefly, cells were vigorously shaken for 30 min at 4 °C and centrifugated at 16,000× *g* for 20 min at 4 °C. Protein (containing in the supernatant) was quantified by Bio-Rad Protein assay kit (Bio-Rad, Hercules, CA, USA). Western blot of samples at 1 mg/mL was made using Jess Simple Western system (We-Met, Lille, France) for all proteins (FAK, ab40795, Abcam, Cambridge, UK; RICTOR, ab176850, Abcam; TUBERIN, ab226800; Abcam). Normalization was made by total protein quantification.

## 3. Results

### 3.1. Non-Specific Phagocytosis Activity of hiPSC-RPE Control and AMD Cells in Basal Condition and under Oxidative Stress Condition

**Figure 1:**
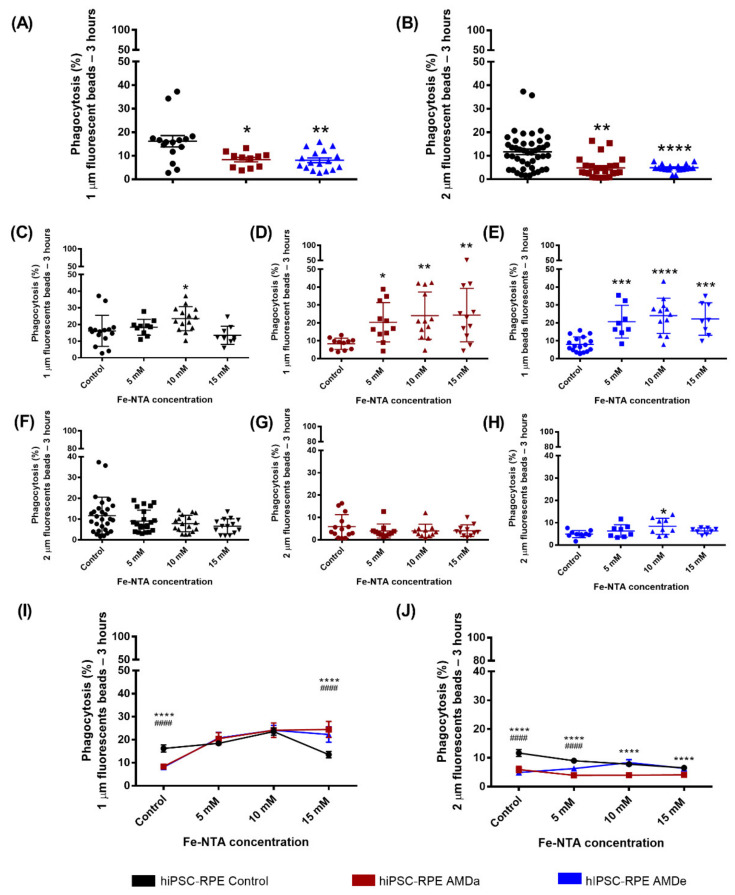
Non-specific phagocytosis function of hiPSC RPE Control and AMDa/e cell lines in both basal and oxidative stress conditions. Non-specific phagocytosis quantification in hiPSC-RPE Control (N = 6, n = 3), AMDa (N = 6, n = 3) and AMDe (N = 3, n = 3) cells in basal condition (**A**,**B**) and 24 h oxidative stress condition induced by different Fe-NTA concentrations (**C**–**J**). For I and J, comparison between hiPSC Control and AMDa (*) or AMDe (#). Statistical analysis: Kruskal–Wallis. * *p* < 0.05 ** *p* < 0.01 *** *p* < 0.001 ****, ^####^ *p* < 0.0001.

hiPSC-RPE cells derived from AMDa and AMDe patients had a lower non-specific phagocytosis activity (8.34 ± 1% and 8.07 ± 1% for 1 µm beads, respectively, 5.93 ± 1.4% and 4.90 ± 1.6% for 2 µm beads, respectively) than hiPSC-RPE Control cells (16.18 ± 2.4% for 1µm beads and 11.65 ± 1.7% for 2 µm beads, respectively) after 3 h of bead exposure (Figure 1A,B). Surprisingly, oxidative stress exposure leads to an increase in the 1 μm beads’ non-specific phagocytosis after 3 h POS exposure in both hiPSC-RPE Control (increased from 16.18 ± 2.4% to 23.53 ± 1.9% with 10 mM of FeNTA) and AMD cells (20.41 ± 3.3% for AMDa and 20.72 ± 3.2% for AMDe, 5 mM of Fe-NTA) (Figure 1C–E). Moreover, a higher 1 µm bead non-specific phagocytosis activity in hiPSC AMDa/e compared to hiPSC-RPE Control was observed (Figure 1I). In contrast, there was no effect of Fe-NTA treatment on non-specific phagocytosis of 2 μm beads for all cell lines after 3 h POS exposure (Figure 1F–H). However, the 2 µm beads’ non-specific phagocytosis activity of hiPSC-RPE Control remained higher than AMDe cells (5 mM) and AMDa cells (5, 10 and 15 mM of FeNTA) (Figure 1J). No difference was observed for 24 h POS exposure in all hiPSC-RPE cell lines under basal and oxidative stress conditions (data not shown).

### 3.2. Specific Phagocytosis Activity of hiPSC-RPE Control and AMD Cells in Basal Condition and under Oxidative Stress Condition

**Figure 2:**
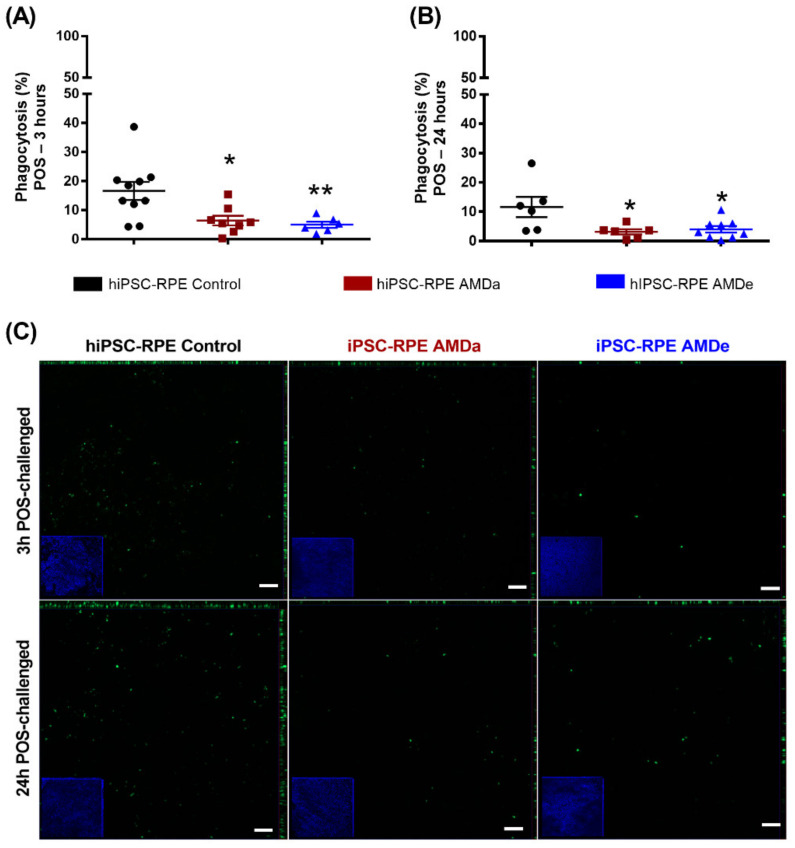
Specific phagocytosis function of hiPSC RPE Control and AMDa/e cell lines in basal condition. Quantification of 3 h POS exposure (**A**) and 24 h POS exposure (**B**) in hiPSC RPE Control (N = 5, n = 2), AMDa (N = 6, n = 2) and AMDe (N = 3, n = 2) cells in basal condition. Representative immunofluorescence of 3 and 24 h POS exposure in hiPSC-RPE Control and AMDa/e cells (**C**). Micrographs on the borders are related to an orthogonal view of hiPSC-RPE cells (N = 2), AMDa (N = 3) and AMDe (N = 3) Scale: 50 µm. Statistical analysis: Kruskal–Wallis. * *p* < 0.05 ** *p* < 0.01.

**Figure 3:**
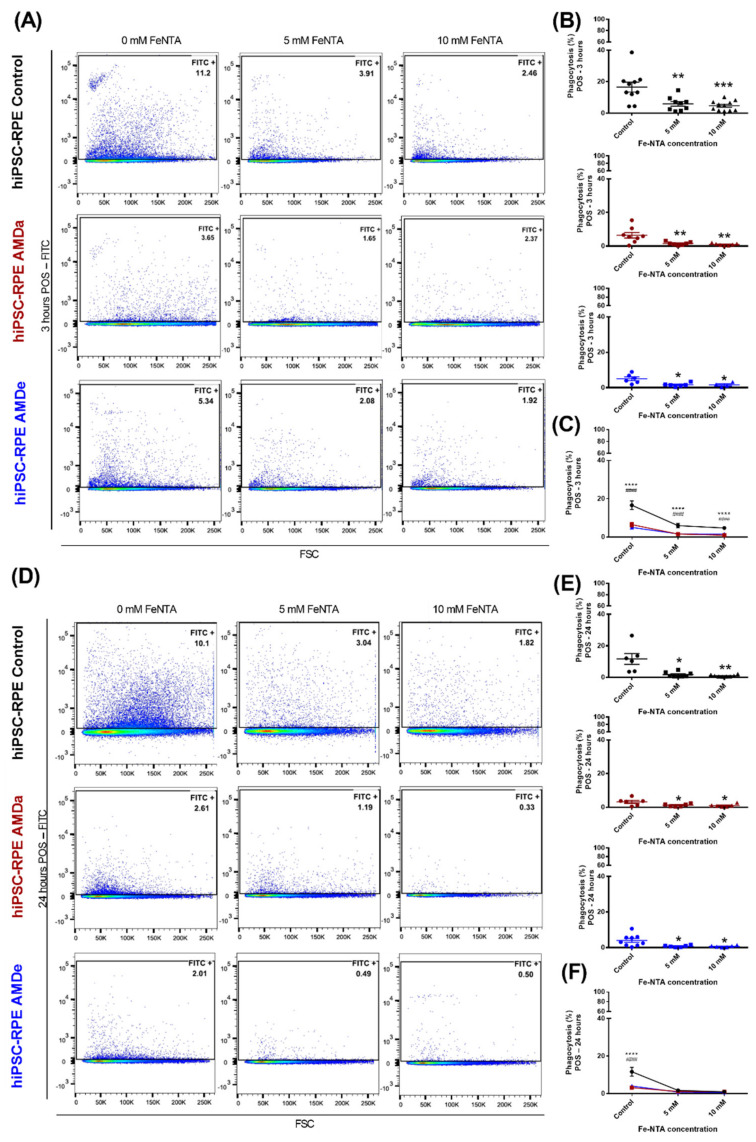
Specific phagocytosis function of hiPSC RPE Control and AMDa/e cell lines in oxidative stress condition induced by Fe-NTA. Flow cytometry analysis (**A**,**D**) and quantification (**B**,**C**,**E**,**F**) of 3 h POS challenged and 24 h POS challenged of hiPSC RPE Control (N = 5, n = 2), AMDa (N = 6, n = 2) and AMDe (N = 3, n = 2) cells in 24 h oxidative stress condition induced by different Fe-NTA concentrations). For C and F, comparison between hiPSC Control and AMDa (*) or AMDe (#). Statistical analysis: Kruskal–Wallis. * *p* < 0.05, ** *p* < 0.01, *** *p* < 0.001, ****^, ####^ *p* < 0.0001.

In basal condition, hiPSC-RPE cells derived from AMDa and AMDe patients had a lower specific phagocytosis activity (6.43 ± 1.7% and 5 ± 1.1%, respectively) than hiPSC-RPE Control cells (16.60 ± 3.1%) after 3 h POS exposure (Figure 2A). Specific phagocytosis was also lower in hiPSC-RPE AMDa and AMDe cells (3.17 ± 0.9% and 6.45 ± 2.2%, respectively) compared to hiPSC-RPE Control cells (13.27 ± 3.7%) after 24 h POS exposure (Figure 2B). We confirmed these results by qualitative immunofluorescence analysis of 3 and 24 h POS challenged with all cell lines. hiPSC-RPE Control cells had more fluorescence POS integration in both time conditions compared to AMD cell lines (Figure 2C). Under oxidative stress, we observed an inhibition of the specific phagocytosis of POS in all hiPSC-RPE cell lines after 3 (Figure 3A,B) and 24 h POS exposure (Figure 3C,D). However, the specific phagocytosis levels of hiPSC-RPE Control during oxidative stress condition remained higher than hiPSC-RPE AMDa/e for 3 h POS exposure (Figure 3C). A contrario, the phagocytosis activity of both hiPSC-RPE Control and AMDa/e cell lines were similar from 5 mM of FeNTA for 24 h POS exposure (Figure 3F). Similar results on phagocytosis activity condition were observed with hiPSC-RPE cells maintained for 2 months in culture in both basal and oxidative stress (data not shown).

### 3.3. Pathways Activated during Specific Phagocytosis Activity in hiPSC-RPE Cells

**Figure 4:**
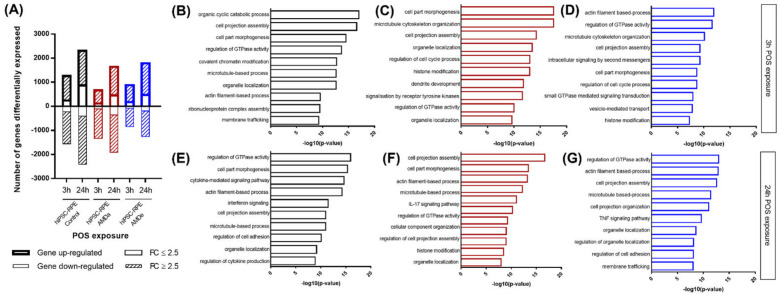
RNA seq analysis of both hiPSC RPE Control and AMD cells after 3 h and 24 h POS exposure. Number of differentially expressed genes in hiPSC-RPE Control (N = 6) AMDa (N = 6) and AMDe cells (N = 3) in basal condition, and after 3 h and 24 h POS exposure (**A**). The Gene Ontology (GO) analyses of highly or lowly (2.5 ≥ FC ≥ 2.5) expressed in hiPSC-RPE Control (**B**,**E**) AMDa (**C**,**F**) and AMDe cells (**D**,**G**) after 3 h (**B**,**C**,**D**) and 24 h (**E**,**F**,**G**) POS exposure.

Gene expression after 3 or 24 h of POS incubation was analyzed by RNA-Seq based transcriptomic profiling. After 3 h of exposure, POS induced modifications of 2349 gene expressions with signals detectable in hiPSC-RPE Control: 783 genes (33.42%) were downregulated and 1098 genes (46.74%) were upregulated with a fold change of 2.5 or more difference. After 3 h POS exposure, we found 1892 genes with differential expression (DE) in hiPSC-RPE AMDa (557 downregulated—29.44% and 1115 upregulated—58.93%) and 1469 in hiPSC-RPE AMDe (541 downregulated—36.83% and 675 upregulated—45.95%) (Figure 3A). Following, 24 h POS exposure induced regulation of 3442 genes in hiPSC-RPE Control: 893 downregulated (25.94%) and 1593 upregulated (46.28%). For hiPSC-RPE AMD, 24 h POS exposure led to modification of 2725 genes for AMDa cell lines (728 downregulated—26.72% and 1185 upregulated—43.49%) and 2380 genes for AMDe cell lines (848 downregulated—35.63% and 860 upregulated—36.13%) (Figure 4A). The top 10 Gene ontology term analysis of this DE gene showed a significant enrichment of cellular function as part morphogenesis, microtubule-based process and regulation of GTPase activity in both 3 h (Figure 4B–D) and 24 h POS exposure (Figure 4E–G).

### 3.4. Difference between hiPSC Control Cells and AMD Cells in the PI3K/Akt, mTor and MEK/ERK Signaling Pathway Related to Specific Phagocytosis Activity

Significant changes in the expression of genes involved in regulation of the GTPase pathway were observed between hiPSC-RPE Control and AMDa/e after 3 and 24 h POS exposure (Table 1).

**Figure 5:**
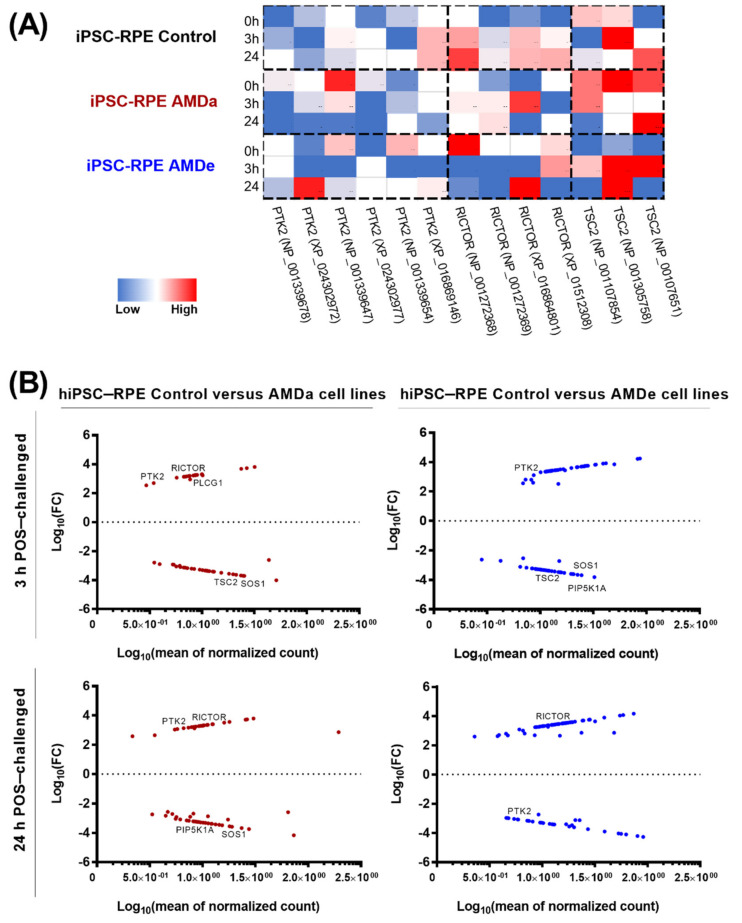
Analysis of specific mTOR/PI3K-AKT/MEK-ERK pathway with RNA-seq. Heat map of 3 enriched genes (*PTK2, TSC2, RICTOR*) involved in GTPase regulation pathway in hiPSC-RPE Control, AMDa and AMDe cells in basal condition and after 3 and 24 h POS exposure (**A**). Volcano plot of differentially expressed genes between hiPSC-RPE Control and AMDa cells and between hiPSC-RPE Control and AMDe cells after 3 and 24 h POS exposure (**B**). hiPSC-RPE Control cells N = 6, hiPSC-RPE AMDa cells N = 6, hiPSC-RPE AMDe N = 3.

**Figure 6:**
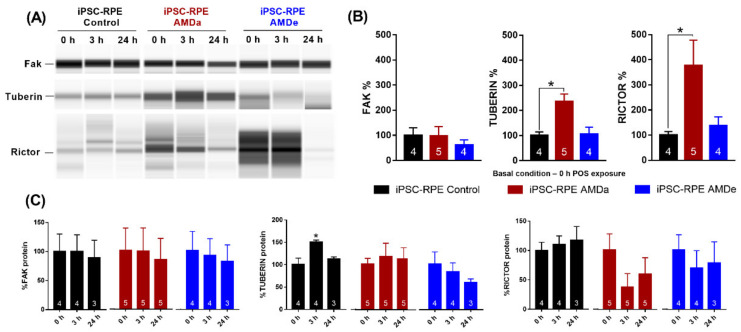
Analysis of expression levels and activity of proteins after POS exposure. Western blot analysis of fak, rictor and tuberin (**A**) and Western blot quantification (**B**,**C**) on hiPSC-RPE Control (N = 4), AMDa (N = 5) and AMDe cells (N = 4) (one analysis per cell line). Statistical analysis: one-way ANOVA and post hoc Turkey’s multiple comparison. Comparison of each dose between hiPSC-RPE Control and AMD cells (**B**) or for each cell line between 0 and 3 h/24 h (**C**); * *p* < 0.05.

Focusing on the regulation of GTPase activity during POS exposure, significant differences in the isoform expression for three major genes involved in phagocytosis regulation (ptk2, tsc2 and rictor) was observed in each cell population (Figure 5A). In the basal condition, rictor was significantly more expressed in hiPSC-RPE AMDa/e compared to hiPSC-RPE Control cells (Figure 5A). Moreover, a higher expression of ptk2 was observed in hiPSC-RPE AMDa (except for NP_001339654) compared to the Control population. No difference of tuberin was observed between all populations in the basal condition. When comparing induced and suppressed genes between all cell lines during 3 h POS exposure, respectively, 46 (41% induced) and 66 (51% induced) genes with a fold change of at least 2.5 were differentially expressed in hiPSC-RPE AMDa and AMDe compared to hiPSC-RPE Control (Figure 5B). After 24 h POS exposure, 50 (44% induced) and 68 (64% induced) genes with a fold change of 2.5 or more difference in hiPSC-RPE AMDa and AMDe, respectively, were differentially expressed (Figure 5B). Changes were observed in genes involved in PI3K/Akt as well as the interconnected mTOR and MEK/EKT signaling pathway. In both AMD cell lines, the expression of ptk2 was lower than in hiPSC-RPE Control cell lines after 3 h POS exposure. After 24 POS exposure, ptk2 expression was lower in hiPSC-RPE AMDa cell lines and higher in hiPSC-RPE AMDe cell lines compared to hiPSC-RPE Control cell. We also found that rictor was highly expressed in hiPSC-RPE Control cells compared to AMD cell lines after both 3 and 24 h POS exposure. Lower expression of tsc2 was observed in hiPSC-RPE Control cells compared to hiPSC-RPE AMDa/e after 3 h POS challenged. At the protein level, differences were observed in the basal condition for tuberin and rictor (along with tsc2 and rictor expression, respectively) but not for fak (ptk2 expression) only in hiPSC-RPE AMDa cells compared to hiPSC-RPE Control cells (Figure 6A,B). No difference was observed between hiPSC-RPE AMDe cells and Control cells for any of these three proteins (Figure 6A,B). We observed an increase in tuberin after 3 h POS exposure only in hiPSC-RPE Control cells compared to the basal situation (Figure 6C). No difference in rictor, fak or tuberin levels was found for hiPSC-RPE AMD cells for 3 and 24 h POS exposure (Figure 6C).

## 4. Discussion

This study investigated phagocytosis activity in hiPSC-RPE cells derived from healthy control subjects, AMDa and AMDe patients in basal condition and under oxidative stress induced by intracellular accumulation of iron. The daily clearance phagocytosis of POS by the RPE is critical for photoreceptor function and vision [26]. Phagocytosis dysfunction of RPE cells has been observed in AMD, but given the state of our knowledge, it is not clear whether this phenomenon is causative or a consequence of AMD [16,22]. Recent studies have shown that RPE phagocytic activity decreased with age, which could be partially explained by lipofuscin and/or iron accumulation into the retina during the aging process [22,27,28]. In this study, we observed that both specific and non-specific phagocytosis activity was altered in hiPSC-RPE AMDa/e compared to hiPSC-RPE Control cells in basal condition without oxidative stress exposure. To the best of our knowledge, our results are the first to demonstrate that phagocytosis activity is dysregulated in a cellular model obtained from hiPSC-RPE cells derived from an AMDa/e patient’s blood cells.

RPE cells recognize the distal part of POS by detection of phosphatidylserine, either directly by CD36, or by Gas6/ProS or MFG-EO for the MERTK receptor [29,30,31]. Receptor tyrosine kinases such as MERTK are known activators of the mTOR pathway [32]. Moreover, initiation of signaling by POS binding results in activation and phosphorylation of intracellular kinases such as Akt, a downstream substrate for PI3K [26]. Implication of the AKT/PI3K pathway in RPE phagocytosis activity was confirmed by ablation of this function after pharmacological inhibition of Akt [29]. By RNA seq and Western blot analysis, we observed differences for three major proteins involved in mTOR/PI3K-AKT/MEK-ERK pathway. Our results suggest that RPE phagocytosis dysfunction observed in hiPSC-RPE AMD could be partially explained by the impairment/dysfunction of genes expression underlying this process.

It is likely that the primary damage leading to AMD occurs at the level of the RPE and is caused by oxidative stress [33], which may partially impair phagocytosis function [14]. In this study, the impact of oxidative stress mediated by Fe-NTA on specific and non-specific phagocytic activity of hiPSC-RPE Control, AMDa and AMDe cells was evaluated. In this context, an inhibition of specific phagocytosis in hiPSC-RPE Control and AMDa/e cells was reported in a similar manner. A contrario, oxidative stress exposure induced a stimulation of 1 µm non-specific phagocytosis in all cell lines. A difference between specific and non-specific phagocytosis has been reported in ARPE-19 cells, an immortalized cell line of RPE cells [14]. POS and polystyrene beads were not internalized by RPE cells in similar numbers. Second, they showed that oxidative stress led to an inhibition of specific phagocytosis of ARPE-19 cells but had no effect on non-specific phagocytosis of fluorescent beads. However, both specific and non-specific phagocytosis were inhibited in oxidative stress mediated by H_2_O_2_ and iron ions. In our study, we observed a strong effect of Fe-NTA treatment on specific phagocytosis activity and lack (2 µm) or reverse (1 µm) effect on non-specific phagocytosis. The stimulation of 1 μm non-specific phagocytosis of polystyrene beads by Fe-NTA exposure may be explained by activation of pathways other than PI3K/AKT/mTOR and MEK/ERK pathways, which are known to be involved in specific phagocytosis. The width of outer segment fragments engulfed by human RPE cells is around 1.2 μm [34,35]. If particles are smaller, they may be internalized though endocytic pathways [36]. Moreover, larger particles remain bound to the RPE surface if phagocytosis does not occur.

In our RNAseq analysis, we focused on gene regulation after 3 and 24 h POS exposure without additional oxidative stress. In this context, we demonstrated that *PTK2,* the gene of focal adhesion kinase (FAK), was statistically less expressed in hiPSC RPE AMDa/e cells compared to control cells after 3 and 24 h POS exposure. FAK plays an important role in transmission of αvβ5-induced cytoplasmic signals and in the reorganization of actin cytoskeleton during POS phagocytosis [37]. We observed no difference in gene expression of FAK by Western blot analysis, with no modification of FAK levels during POS incubation.

The phagocytic challenge, by activating FAK at the apical RPE surface, leads to subsequent activation of phagocytosis effectors through the PI3K/AKT/mTOR and MEK/ERK pathway such as *TCS2* and *RICTOR*. In our study, we observed higher upregulation of *RICTOR* in hiPSC-RPE Control cells after POS challenged compared to both hiPSC-RPE AMDa/e cell lines. Involvement of rictor was confirmed by overexpression of *RICTOR* in hiPSC-RPE AMDa in basal condition compared to hiPSC-RPE Control cells. Rictor is a component of mTORC2, one of the two complexes comprising the mTOR pathway, and alteration of the mTOR signaling network has been observed in senescent RPE cells [38].

Knockdown of rictor leads to defective actin organization, a process essential to formation of a classical phagocytic cup [19,39]. mTORC2 also contributes to the recruitment of F-actin and Rac1 activation, a Rho GTPase that affects the local assembly or disassembly of filamentous F-actin [40,41]. Reduction of *Rac1* expression inhibits POS internalization by RPE cells [19,40]. Rictor contributes to regulation of the actin cytoskeleton through *TSC2* inhibition and activation of Rho GTPases [39,42]. In our study, we found that *TSC2* is upregulated in hiPSC-RPE AMDa/e cells compared to hiPSC-RPE Control cells after 3 h POS challenged. Overactivation of TSC2 can contribute to aberrant mTOR signaling, which is involved in aging and other disease such as AMD [43]. A more activated mTOR signaling pathway could explain why hiPSC-RPE AMDa cells were more sensitive to oxidative stress than control cells [10]. The mTOR network has emerged as one of the main pathways involved in mammalian cells as RPE [38]. mTOR inhibition could be a future promising therapeutic target to slow down the pathological processes occurring in RPE cells in AMD.

## 5. Conclusions

In conclusion, this study confirmed phagocytosis dysfunction in AMD in a cellular model based on an hiPSC-RPE-derived AMDa/e patient. This observation raised the question of whether RPE phagocytosis dysfunction is a cause or a consequence of AMD. Our data support the hypothesis that (i) oxidative stress may contribute to impairment of RPE phagocytosis; (ii) a specific phagocytosis pathway should be more deeply explored in order to identify new potential therapeutic targets for AMD.

## Figures and Tables

**Table 1:** Comparison between hiPSC-RPE Control (N = 6), AMDa (N = 6) and AMDe (N = 3) cells of selected Gene Ontology biological processes involved in 3 and 24 h POS challenged.

Comparison between hiPSC-RPE Control, AMDa and AMDe Cells of Selected GO Biological Process Involved in 3 h and 24 h—POS Challenged				
POS-Challenged Time	3 h		24 h	
hiPSC-RPE Cell Lines	Control vs. AMDa	Control vs. AMDe	Control vs. AMDa	Control vs. AMDe
GO Biological Process	Log_10_(*p*-value)			
Regulation of GTPase activity	−9.474354469	−17.7943	−12.0125	−13.1224
Cell junction organization	−4.06929	−7.56126	−3.61127	−7.53024
Cell part morphogenesis	−7.300745101	−16.7688	−4.77497	−9.28681
Microtubule-based process	−6.04778	−10.1841	−6.04778	−9.19358
Plasma membrane bounded cell projection assembly	−7.26267	−11.7729	−7.26267	−8.91932
Organelle localization	−4.66769	−14.0398	−4.7191	−10.326
Membrane trafficking	−4.7262	−12.4136	−3.62451	−5.06347

## Data Availability

The original contributions generated for this study are included in the article; the data presented in this study are available on request from the corresponding author.
